# Fresh-State and Mechanical Properties of High-Performance Self-Compacting Concrete with Recycled Aggregates from the Precast Industry

**DOI:** 10.3390/ma12213565

**Published:** 2019-10-30

**Authors:** Tiago Barroqueiro, Pedro R. da Silva, Jorge de Brito

**Affiliations:** 1 Instituto Superior Técnico, Universidade de Lisboa, 1649-004 Lisbon, Portugal; tiagobarroqueiro87@gmail.com; 2 CERIS, Instituto Superior de Engenharia de Lisboa, Instituto Politécnico de Lisboa, 1500-310 Lisbon, Portugal; silvapm@dec.isel.pt; 3 CERIS, Instituto Superior Técnico, Universidade de Lisboa, 1649-004 Lisbon, Portugal

**Keywords:** high-performance self-compacting concrete, coarse and fine recycled aggregates from the precast industry, fresh properties, mechanical properties

## Abstract

The urgent need to change the less positive impacts of the construction industry on the environment, and more specifically the production and use of concrete, is the main motivation for the research for more efficient and environmentally sustainable solutions. This paper presented the results of an experimental campaign whose ultimate goal was to produce high-performance self-compacting concrete (SCC) using recycled aggregates (RA) from the precast industry. The results of the fresh-state and mechanical properties tests performed on six concrete mixes (using RA from the precast industry) were presented. The first concrete mix is a reference mix using natural aggregates only (100% NA), and the remaining five mixes had various contents of fine (FRA) and coarse (CRA) recycled aggregates in concrete’s composition: (2) 25/25% (25% RA); (3) 50/50% (50% RA); (4) 100/100% (100% RA); (5) 0/100% (100% CRA); (6) 100/0% (100% FRA). The results showed that the high-performance concrete mixes with RA from the precast industry performed worse than the reference mix. However, taking into account all the mechanical properties studied, it can be concluded that RA from precast concrete elements are of very good quality and can be incorporated in the production of high-performance SCC. The potential demonstrated by the combined use of fine and coarse recycled aggregates was also emphasized. This type of work is expected to effectively contribute to raise awareness among the various players in the construction industry, particularly in the precast concrete industry, to the feasibility of using RA in significant quantities (notably coarse aggregates) and to the safety needed to assume structural functions, even for applications where high performance is required.

## 1. Introduction

### 1.1. Background

The issue of self-compacting concrete (SCC) with recycled aggregates (RA) is of great relevance to the community, which increasingly demands that the construction industry adopts new processes that minimize negative impacts on the environment. SCC with RA is a technical solution that can achieve precisely those objectives.

The use of RA in concrete production has a great environmental benefit through savings in natural aggregate (NA) extraction and the elimination of waste, resulting from the demolition of obsolete structures or, in the particular case of this study, from the waste from the precast industry.

The properties of RA are clearly influenced by the quality of the original concrete, especially the quality and quantity of mortar adhered to its surface. The quality of the mortar depends on the water/binder ratio used in the original concrete and its quality depends on the strength of the concrete and the crushing method used [1]. In this sense, RA from the precast industry usually have a higher quality compared to those obtained from construction and demolition waste (CDW). This is mainly due to the quality of the original precast concrete that usually has compressive strength values above 40–50 MPa and lower water–cement ratio, among others.

One of the main advantages of SCC is its ability to flow and compact only under the action of its own weight, filling the formwork with its reinforcement, tubes, negative, etc., to maintain the homogeneity [2]. Therefore, this type of concrete does not need to be vibrated (unlike conventional concrete), thus promoting a great environmental benefit, particularly in terms of energy saving and no noise during casting [3].

In conventional concrete, it is necessary to apply additional energy to vanquish the internal friction between the particles to allow them to mix and fill the molds correctly. However, in an SCC, this does not have to be done because the concrete itself achieves this effect. This is reflected in the fresh-state tests and is achieved by optimizing the mix composition and incorporating admixtures and superplasticizers.

The specific requirements for fresh SCC depend on the type of application, with particular emphasis on confinement conditions (concrete element geometry, quality, type, and location of reinforcement, among others), concrete laying equipment, concrete laying methods, and surface finishing methods [3].

The properties to be evaluated in the fresh-state test may be characterized by the following characteristics: Fluidity, flow velocity with or without obstructions, filling ability, flow ability, passing ability, and segregation resistance.

High-performance concrete (HPC) is currently used only in particular situations, such as in tall buildings and structures located in particularly harsh environments [4]. Given the need to flow in densely reinforced areas, it is sometimes necessary for this type of concrete to have self-compacting characteristics. This context justifies the need for high-performance self-compacting concrete (HPSCC). In fact, in situations where higher workability and better resistance to both mechanical loading and chemical attack may be required, HPSCC may be used [5]. The use of this type of concrete allows the structures to have a lifetime of 100 years or more [4].

The most relevant and enabling component of HPC production is silica fume. This allows improvements in mechanical properties, such as modulus of elasticity, flexural strength, and compressive strength, among others [6]. In addition, silica fume contributes to HPC’s increased durability [7,8]. Silica fume has a greater impact on concrete compressive strength during the first 28 days. After 28 days, the concrete will gradually continue to gain strength, although the strength gaining rate is much slower [6]. The amount of silica fume to be used in HPC ranges from 10% to 15% in cement weight [7,9]. If a small amount of silica fume is incorporated (less than 5%), its use will not be efficient [10]. On the other hand, for amounts greater than 15%, the space between the cement particles will not be sufficient to accommodate all the silica, a part of which is thus wasted [11].

HPC consist of cement, water, fine and coarse aggregates, superplasticizer, and/or mineral additions (e.g., fly ash, blast furnace slag, and silica dust, among others) [12,13]. They usually use CEM I cement, class 42.5 or 52.5, in large quantities of around 400–550 kg/m^3^ with water–binder ratios between 0.25 and 0.35 [10,14]. This concrete has higher compressive strength than conventional concrete. Despite this, strength is not always the main property required. However, due to the high amounts of cement, HPC has higher environmental impacts than conventional concrete. Thus, in the context of sustainability in the construction industry, it is important to create a set of strategies regarding its use. Although there is no well-defined line of separation between conventional concrete and HPC, ACI 211.4R [15] characterizes HPC as concrete with a compressive strength greater than 41 MPa. NP EN 206-1 [2] considers that, to be classified as HPC, a concrete must have a strength class greater than C50/60.

The use of RA in the production of HPSCC is still incipient and very little work is available on the subject. Considering the particular use of RA from the precast industry, it was almost impossible to find scientifically based references. However, it was possible to find some published work on some of the main topics of this paper. For example, Farhad et al. [16] performed interesting work on developing HPSCC using RA and rubber granulate. The authors stated that the addition of RA did not significantly compromise its mechanical strength, thereby raising the produced mixes to a high-performance level.

Similarly, Grdic et al. [17] performed work on the properties of SCC prepared with coarse recycled aggregates (CRA) and noted minor losses in strength. On the other hand, Corinaldesi and Moriconi [18] studied the joint use of CRA and FRA in the production of SCC, concluding that while the use of fine recycled aggregates (FRA, used to replace sand) negatively affected compressive strength, the latter remained unchanged when CRA was used as a replacement of coarse natural aggregates (CNA). In the same sense, Santos et al. [19], who conducted a study on the mechanical performance evaluation of SCC with FRA and CRA from the precast industry, concluded that the RA used performed better than reported in similar works. They also concluded that 25% RA, 50% RA, and 100% CRA mixes exhibited satisfactory mechanical and durability behavior for the production of concrete with structural functions.

### 1.2. Objectives

Because aggregates account for 55–80% of the concrete volume [5], this study focused on the use of RA from the precast industry replacing NA, which not only saves natural resources, but also frees landfill space. In addition, this study included substitute/complementary materials for cement the byproducts of silicon (silica fume) and coal burning for energy production (fly ash).

In this sense, six HPSCC mixes were produced with the following incorporation proportions (FRA/CRA %): 0/0%; 25/25%; 50/50%; 100/100%, 0/100%; 100/0%.

To evaluate the fresh-state properties, the following tests were performed: The slump-flow test, V-funnel test, L-box test, j-ring test, and the sieve segregation test.

To evaluate the mechanical properties, the following hardened state tests were performed: Density of hardened concrete, compressive strength, splitting tensile strength, secant elastic modulus of elasticity, ultrasonic pulse velocity test, abrasion resistance, creep and total shrinkage strain.

RA comes from crushing precast concrete parts with a compressive strength class of 65 MPa. RA were subjected to two crushing processes, a primary grinding followed by a secondary grinding, which is similar to what is done for NA.

The percentage of cement substitution with mineral additions (f_ad_), which included limestone filler (LF) and fly ash (FA), was kept constant in all the mixes. A f_ad_ value of 35% was considered, with 5% corresponding to LF and 30% to FA. The amount of silica fume and cement was also kept constant in all mixes: 10% of silica fume and 450 kg/m^3^ of cement. The w/c ratio of all mixes produced was 0.44.

In this paper, the results of the experimental campaign with HPSCC with RA were presented. In addition, HPSCC and RA were analyzed and discussed, and a comparison with similar studies was performed. In the analysis of these properties, the influence of RA’s incorporation in SCC was evaluated. Also, the experimental results were compared with the models predicted in Eurocode 2 [20].

## 2. Materials and Methods

### 2.1. Materials

**Figure 1:**
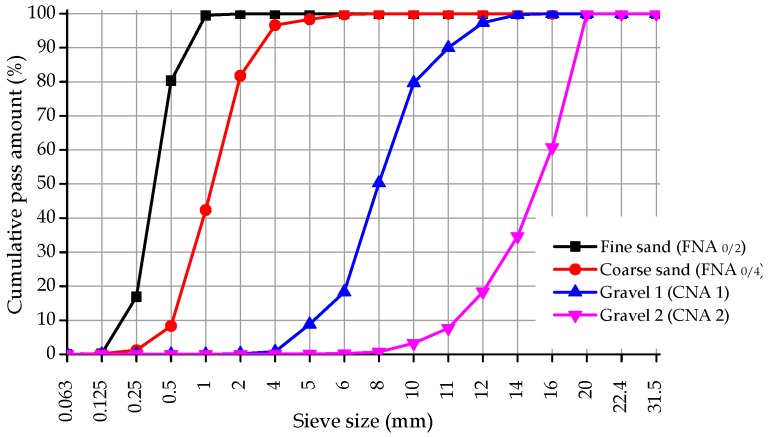
Particle size distribution of the natural aggregates [19].

To carry out the experimental campaign, the following materials were selected:

— Type I cement (CEM I 52.5 R) produced according to NP EN 197-1 [21], with a density of 3200 kg/m^3^, whose properties are shown in Table 1 and Table 2;
— Three mineral additions: Fly ash (FA), produced according to NP EN 450-1 [22] and NP EN 450-2 [23]; limestone filer (LF), produced according to LNEC-E466 [24]; and silica fume (SF), produced according to NP EN 13263-1 [25]. FA, LF, and SF a density corresponding to 2300 kg/m^3^, 2720 kg/m^3^, and 2010 kg/m^3^, respectively, and their properties are shown in Table 1 and Table 2;
— Two limestone coarse aggregates and two siliceous sands observing NP EN 12620 [26], namely: Gravel 1 (density = 2640 kg/m^3^; water absorption = 1.60%; D_max_ = 11 mm), gravel 2 (density = 2690 kg/m^3^; water absorption = 0.80%; D_max_ = 20 mm), coarse sand reference 0/4 (density = 2670 kg/m^3^; water absorption = 0.40%), and fine sand reference 0/2 (density = 2670 kg/m^3^; water absorption = 0.40%). The particle size distribution is presented in Figure 1;
— RA from crushed concrete elements from precast industry of strength class 65 MPa: One coarse recycled aggregate (CRA) (density = 2490 kg/m^3^; water absorption = 2.20%), and one fine recycled aggregate (FRA) (density = 2450 kg/m^3^; water absorption = 7.5%). Since the RA were used in substitution of NA, its particle size distribution is the same;
— A water-reducing admixture (S_p_), produced observing NP EN 934-1 [27] and NP EN 934-2 [28] (a superplasticizers in liquid form with a density = 1070 kg/m^3^);
— Tap water, which complied with NP EN 1008 [29].

### 2.2. Mix Proportions

SCC are made of coarse aggregates dispersed in a mortar matrix. Thus, once the properties of the mortar matrix suitable for obtaining SCC are defined, it becomes possible to separate the study of the mortars phase from the study of the concrete phase. A first justification for the separate analysis of mortars lies in the greater ease of execution compared to concrete. This facility results in the possibility of testing smaller volumes and faster mortar execution and testing.

Thus, the methodology followed in the present work was based on the method proposed by Nepomuceno [31,32], i.e., prior to the production of SCC, a previous study was performed on mortars to assess the parameters related to this phase (definition of water and superplasticizer volumes). After the mortar were designed, concrete was designed by defining the amount of coarse aggregate. Accordingly, the methodology recommended by Nepomuceno [31,32] was adopted, as has been done in numerous research papers [33,34,35,36,37,38].

Table 3 shows the mix proportions of all studied mixes.

It was necessary to make some choices regarding the various mix parameters, namely:

- It was necessary to set a value for the ratio, in absolute volume, between the total amount of fine materials (cement and mineral admixtures) and of fine aggregates in the mix (V_p_/V_s_), which should preferably be between 0.65 and 0.80 according to Nepomuceno [31,32]. V_p_/V_s_ = 0.80 was chosen using the elements available in the study of Silva and de Brito [35];
- Taking into account the desired strength, the percentage of cement substitution with mineral admixtures (f_ad_) was fixed. Thus, all the studied concrete mixes contemplated the incorporation of LF and FA (in substitution of cement). A f_ad_ value of 35% was considered, with 5% corresponding to LF and 30% corresponding to FA;
- In all mixes, an absolute volume of cement V_c_ = 0.137 m^3^/m^3^ and a percentage of SF relative to the cement mass of 10% (corresponding to V_Sf_ = 0.0137 m^3^/m^3^) were considered;
- V_w_/V_p_ (ratio, in absolute volume, between the total amount of water and that of fine materials) and S_p_/p% (percentage, in mass, of superplasticizer and fine materials in the mix) were kept constant (V_w_/V_p_ = 0.92 and S_p_/p% = 1.24);
- RA came from the crushing of precast elements with a 65 MPa compressive strength class. RA were subjected to two crushing processes, a primary grinding followed by a secondary grinding, similar to what is done for NA.

### 2.3. Test Methods

#### 2.3.1. Fresh-State Tests

Fresh-state tests were performed according to the parameters defined in NP EN 206-9 [39]. Namely, to assess the filling ability, the slump-flow test [40] and the V-funnel test [41] were used; for passing ability’s evaluation, the L-box test [42] and the J-ring test [43] were used; and to assess resistance to segregation, the sieve segregation test was used [44].

#### 2.3.2. Hardened-State Tests

The hardened concrete density test was performed according to NP EN 12390-7 [45] in 150-mm cubic edge specimens. For each reference and age, three specimens were molded and tested at 7, 28, and 91 days.

The compressive strength test was performed according to NP EN 12390-3 [46]. The test was carried out on 150-mm cubic edge specimens, as well as 150-mm-diameter and 300-mm-high cylindrical specimens. For each reference and age, three specimens were molded and tested at 7, 28, and 91 days.

The splitting tensile strength test was performed according to the methodology specified by NP EN 12390-6 [47]. The test was carried out on 150-mm-diameter and 300-mm-high cylindrical specimens. For each reference and age (7, 28, 91 days), three specimens were molded and tested.

The secant elastic modulus of elasticity test was performed according to the LNEC-E 397 [48]. The test was carried out on 150-mm-diameter and 300-mm-high cylindrical specimens. For each reference and age (7, 28, 91 days), three specimens were molded and tested.

The ultrasonic pulse velocity test was performed according to NP EN 12504-4 [49] by testing the same specimens used for compressive strength at the same ages (7, 28, and 91 days).

Abrasion resistance was tested using a grinding wheel. Three specimens (71 mm × 71 mm × 40 mm) were tested according to DIN 52108 [50] at 91 days.

The total shrinkage strain was measured in two prismatic specimens (100 mm × 100 mm × 500 mm) for each concrete reference for 182 days (daily up to 14 days and weekly between 14 days and 182 days) according to LNEC specification E 398 [51].

Three prismatic specimens (100 mm × 100 mm × 500 mm) were mounted upright on creep frames at 28 days of age and a constant load was applied. Creep strain over time was evaluated every day for 91 days. The test was performed according to LNEC specification E 399 [52].

## 3. Results and Discussion

### 3.1. Fresh-State Properties

In this subchapter, the results obtained in the fresh HPSCC tests were analyzed.

#### 3.1.1. Slump-Flow Test

**Figure 2:**
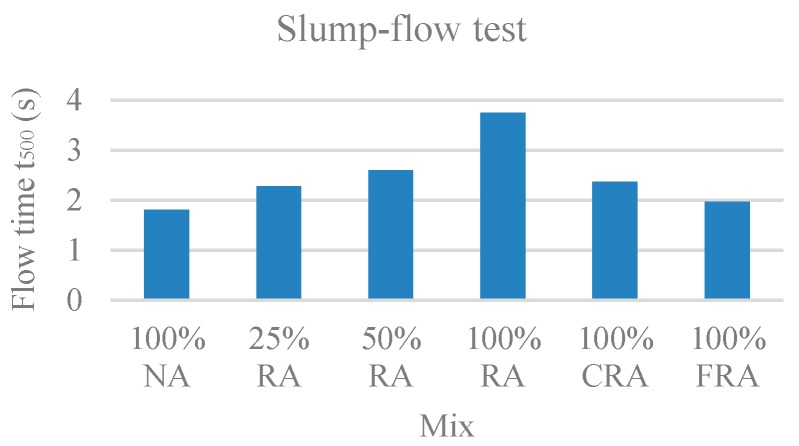
Flow time (t_500_) test results.

**Figure 3:**
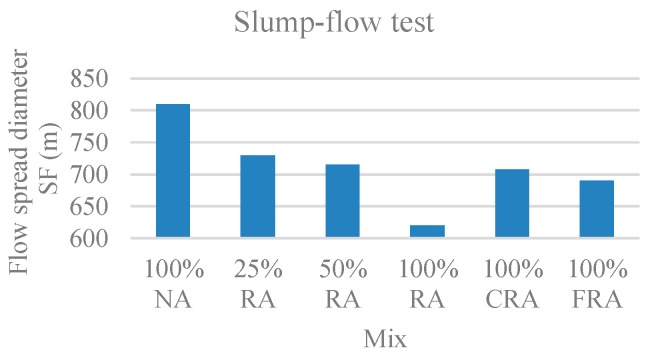
Slump-flow (SF) test results.

The parameters evaluated were the time taken by the SCC to reach a circle of 500 mm, called flow time (t_500_), and the slump-flow diameter (SF). Figure 2 and Figure 3 show the results obtained for t_500_ and SF, respectively.

Through joint observation of Figure 2 and Figure 3, in the mixes 100% NA, 25% RA, 50% RA, and 100% RA, there was an increase in flow time (Figure 2) and a decrease in flow spread diameter (Figure 3). This is justified by the higher absorption of RA compared to NA, given that, for higher RA substitution ratios, a larger amount of mixing water was probably absorbed by the aggregates. Grdic et al. [17] and Kebaïli et al. [53] confirmed these trends.

In terms of both flow time (t_500_) and flow spread diameter, the results obtained by all mixes can be considered satisfactory. In general, there was a good coarse aggregate distribution without any exudation or segregation phenomenon. Coarse aggregates were visible even at the flow spread limit.

According to the classification given by NP EN 206-9 [39], all mixes fell into class VS2 (VS ≥ 2 s) except 100% NA, which had the shortest flow time, belonging to class VS1 (VS ≤ 2 s).

As for the flow spread diameter, the 25% RA, 50% RA, 100% CRA, and 100% FRA mixes fell into the slump-flow class SF2 (660 mm ≤ SF ≤ 750 mm). The 100% NA mix had the largest flow spread diameter, belonging to slump-flow class SF3 (760 mm ≤ SF ≤ 850 mm). On the other hand, 100% RA had the smallest flow spread diameter, belonging to slump-flow class SF1 (550 mm ≤ SF ≤ 650 mm).

#### 3.1.2. V-Tunnel Test

**Figure 4:**
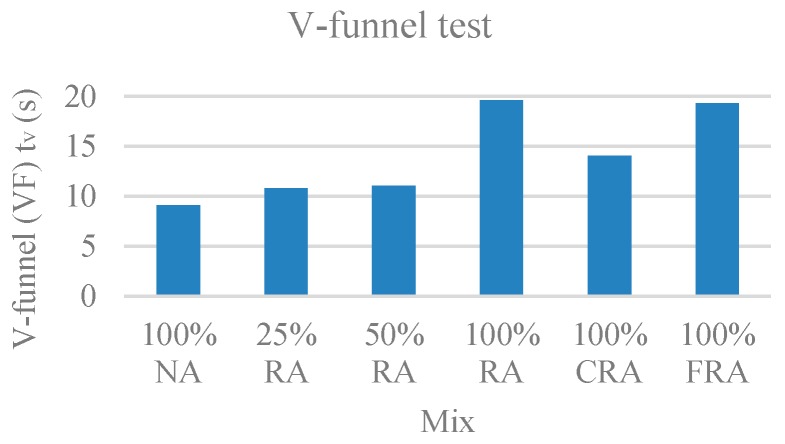
V-funnel (T_v_) test results.

The time that the sample takes to fully flow through the V-funnel (T_v_) was the parameter evaluated in this test. The results are presented in Figure 4.

It was found that the flow time through the V-funnel increased with the percentage of RA. This trend is justified by RA’s higher water absorption and its rougher surface compared to that of NA [54].

For all mixes, the values obtained in the flow time through the V-funnel fit those referred in NP EN 206-9 [39], ranging generally between 9 s and 20 s. Excluding the 100% NA mix, all mixes fell into class VF2 (9 s ≤ VF ≤ 25 s). The mix with 100% NA had the shortest flow time, and the only mix belonging to class VF1 (VF < 9 s).

Visual observation allowed for the checking of the non-blockage effect of coarse aggregate in the V-funnel narrow passage. No exudation was observed. After the test, concrete continued to appear as a uniformly distributed mass.

#### 3.1.3. L-Box Test

**Figure 5:**
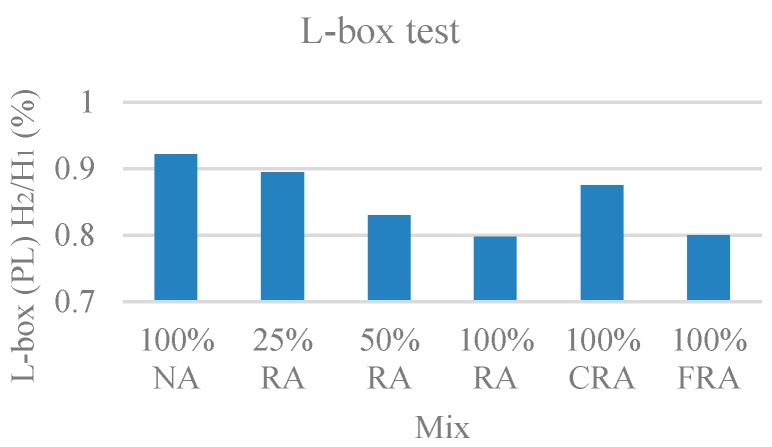
L-box test results.

In this test, the passing ability index in the L-box (PL) was evaluated. Figure 5 presents the results obtained.

It was found that the passing ability index decreased with the incorporation of RA. This was due to the higher water absorption of CRA compared to NA. This trend was also found by Kebaïli et al. [53].

The average values obtained are close, and no exudation or segregation was observed. For this test, the NP EN 206-9 [39] reference value for H_2_/H_1_ was greater than 80%. Therefore, the results obtained fell within this limit, ranging from 80% to 92%. Therefore, all mixes belonged to class PL2.

#### 3.1.4. Sieve Segregation Test

**Figure 6:**
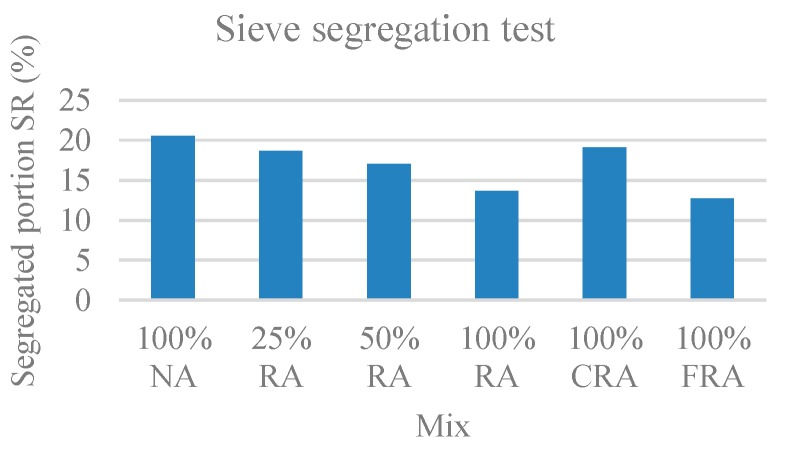
Sieve segregation test results.

In this test, the segregated portion (SR) was evaluated, which corresponds to the proportion of the sample passing through the sieve, relative to the total amount. The results are presented in Figure 6.

The segregation index tended to decrease with the incorporation of RA. This was due to the higher water absorption of RA compared to NA. A similar trend was found in the study of Grdic et al. [17].

These results were within the values referred in NP EN 206-9 [39], i.e., always below 20%.

The mixes with 100% RA and 100% FRA fell into the SR2 class (SR ≤ 15%), corresponding to a smaller tendency toward segregation. The remaining mixes fell into class SR1 (SR ≤ 20%).

#### 3.1.5. J-Ring Test

**Figure 7:**
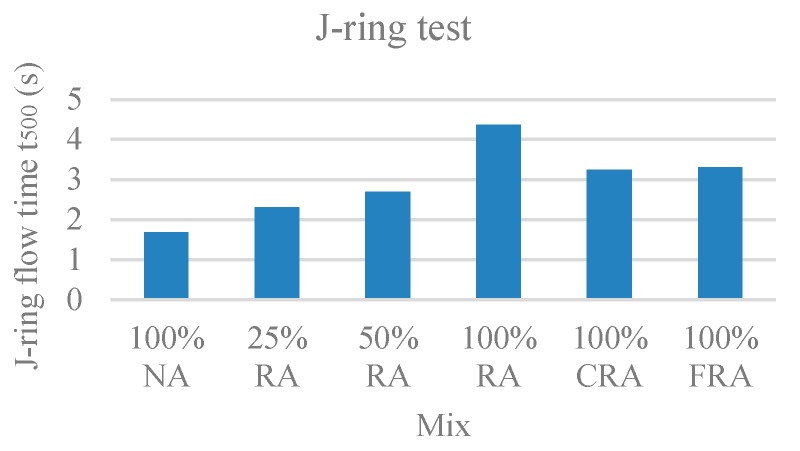
J-ring flow time (t_500_) test results.

**Figure 8:**
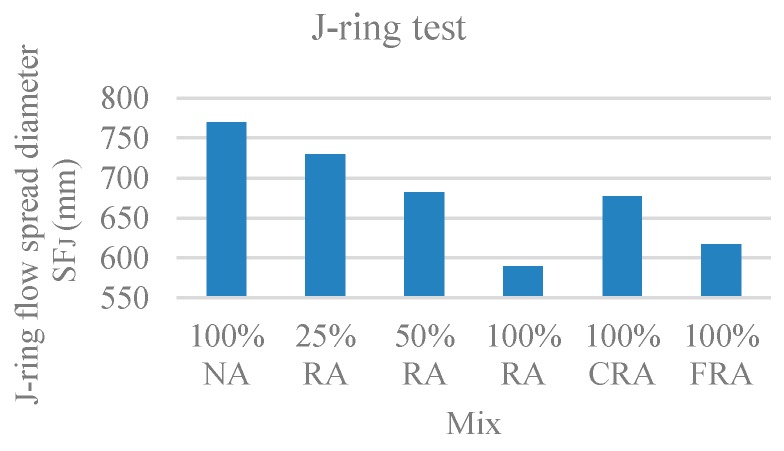
J-ring flow spread diameter test results.

**Figure 9:**
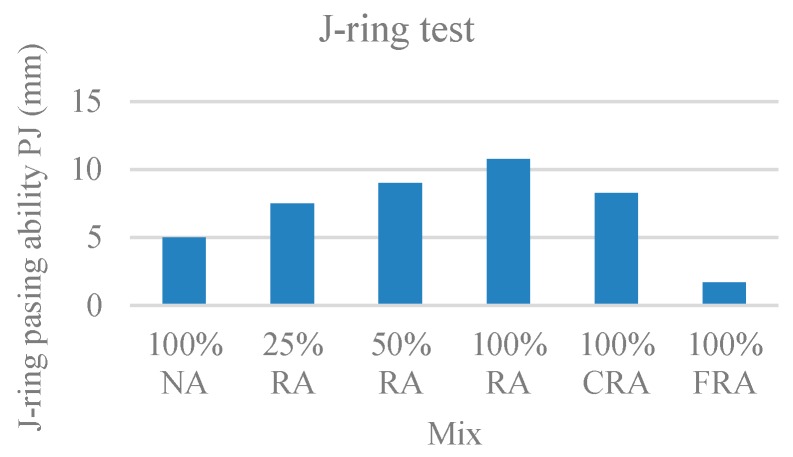
J-ring passing ability PJ test results.

In this test, the time it takes SCC to reach a 500-mm circle (T_500_), the J-ring slump flow diameter (SF_J_), and the ring passing ability (PJ), which corresponds to the blocking gap, were evaluated. Figure 7, Figure 8, and Figure 9 show the results obtained for T_500_, SF_J_, and PJ, respectively.

**Figure 10:**
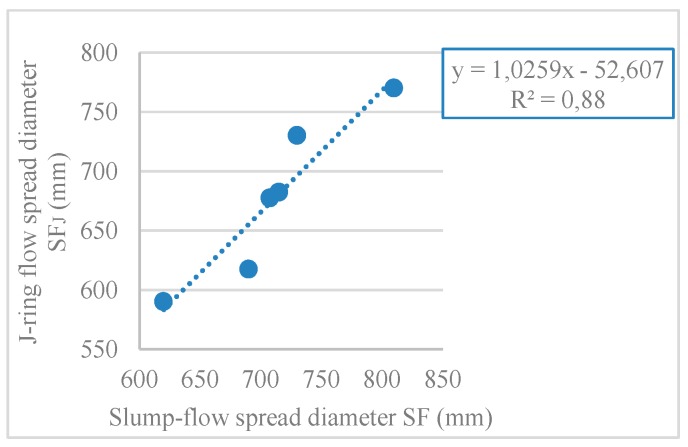
J-ring flow spread diameter SF_J_ and slump-flow spread diameter SF correlation.

Observing the results, with the incorporation of RA, the flow time increased (Figure 7), the J-ring flow spread decreased (Figure 8), and the passing ability increased (Figure 9). The results of the J-ring spread diameter (SF_J_) and the slump-flow diameter (SF) results were strongly correlated (ratio of 0.88), with a linear relationship (Figure 10). This means that both the slump-flows (SF_J_ and SF) vary similarly with the RA content, i.e., the fluidity and passing ability were directly interconnected. Safiuddin et al. [55] found a similar trend, obtaining a 0.99 correlation coefficient.

According to NP EN 206-9’s [39] classification, the 100% NA, 25% RA, and 50% RA mixes belonged to spreading class VJ1 (VJ < 3). The remaining mixes belonged to spreading class VJ2 (VJ ≥ 3).

Regarding the spreading diameter, the 100% NA and 25% RA mixes belonged to spreading class SF_J_3. The 50% RA, 100% CRA, and 100% FRA mixes belonged to the SF_J_2 class. Only the 100% RA mix belonged to SF_J_1 class.

The j-ring passing ability values (PJ) were generally found in class PJ2 for all studied mixes, with values below 10 mm, which correspond to good flowability.

### 3.2. Hardened State Properties

In this subchapter, the results obtained in the hardened SCC were analyzed, namely: Density, compressive strength, splitting tensile strength, modulus of elasticity, ultrasonic pulse velocity, abrasion resistance, shrinkage, and creep.

#### 3.2.1. Density

**Figure 11:**
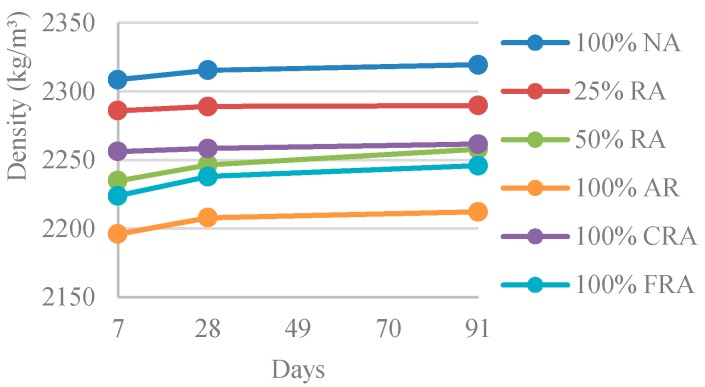
Density at 7, 28, and 91 days.

Table 4 and Figure 11 show the results obtained in the density test.

It was found that the density slightly increased with age (average increase of 8% from 28 days to 91 days). This is justified by the longer curing period, which incorporated more water into the sample, increasing its mass.

Analyzing the relative position of the mixes shown in Figure 11, it appears that density decreased with the incorporation of RA, given the lower density of RA compared to NA’s. The highest density loss was found in the 100% RA mix (5% loss from the reference SCC). A similar trend was found in the work of Pereira-de-Oliveira et al. [54], where the authors obtained a 3% density loss (100% RA concrete) compared to the reference SCC.

#### 3.2.2. Compressive Strength (15 cm × 15 cm × 15 cm Cubes)

**Figure 12:**
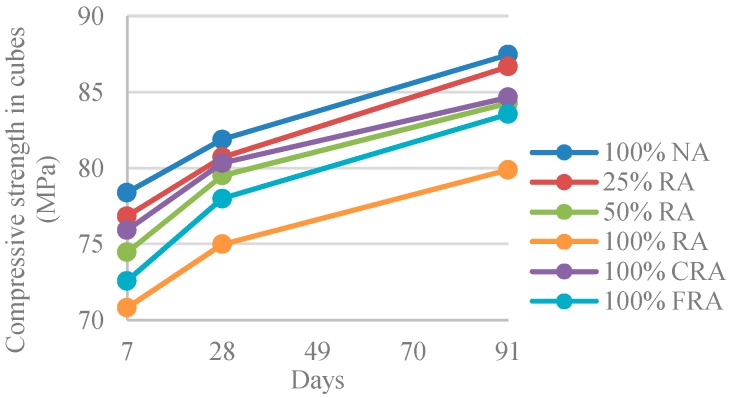
Compressive strength (15 cm × 15 cm × 15 cm cubes) at 7, 28, and 91 days.

Table 5 and Figure 12 show the results of compressive strength in cubes with 15 cm × 15 cm × 15 cm (*f_cm,c_*).

All mixes developed strength rapidly up to 7 days, reaching, on average, about 89% of the strength at 91 days. This is justified by the presence of LF [17] and of SF [6].

Figure 12 shows that replacing NA with RA caused compressive strength decreases from the reference SCC (100% NA) by 1–9%. The compressive strength differences were similar at 7, 28, and 91 days. This is explained by the poorer quality of RA due to their adhered mortar that is responsible for increased aggregates’ porosity and cracking, weakening the transition zone connections between RA and the new binder [56].

The same trends have been found by several authors [17,54,57,58]. Grdic et al. [17] and Pereira-de-Oliveira et al. [54] obtained a compressive strength reduction of 8% and 5%, respectively, comparing the SCC with NA only and the SCC with 100% RA (at 28 days).

**Figure 13:**
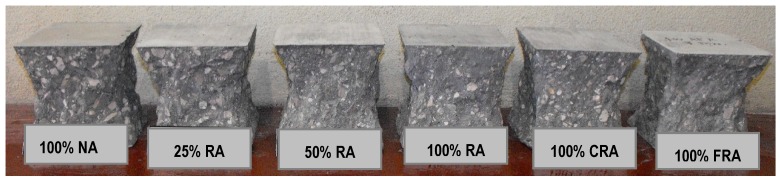
Failure surfaces presented by the different concrete mixes at seven days.

**Figure 14:**
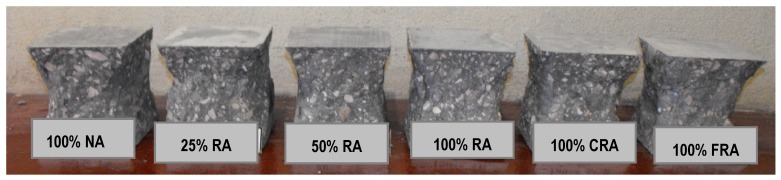
Failure surfaces presented by the different concrete mixes at 28 days.

**Figure 15:**
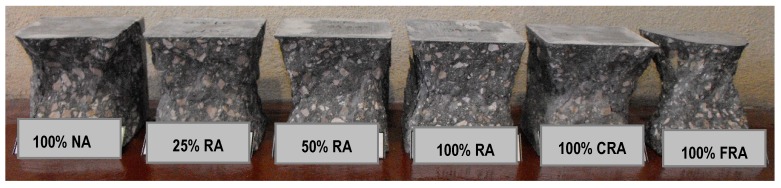
Failure surfaces presented by the different concrete mixes at 91 days.

Analyzing the failure surfaces in the cubic specimens due to uniaxial compression, they showed satisfactory failures (Figure 13, Figure 14 and Figure 15) according to NP EN 12390-3 [36]. The type of failure obtained (explosive failure) is normal in HPSCC, according to Parande [7].

The compressive strength results showed that the reference mix (100% NA) with no RA presented higher compressive strength, reaching 82 MPa and 87 MPa at 28 and 91 days, respectively. The 100% RA mix had the lowest compressive strength of 75 MPa and 80 MPa at 28 and 91 days, respectively (8% reduction when compared to the reference mix).

Thus, according to the results at 28 days, the criteria defining HPC were met (according to ACI 211 [15], which defines HPC as having compressive strength greater than 41 MPa).

**Figure 16:**
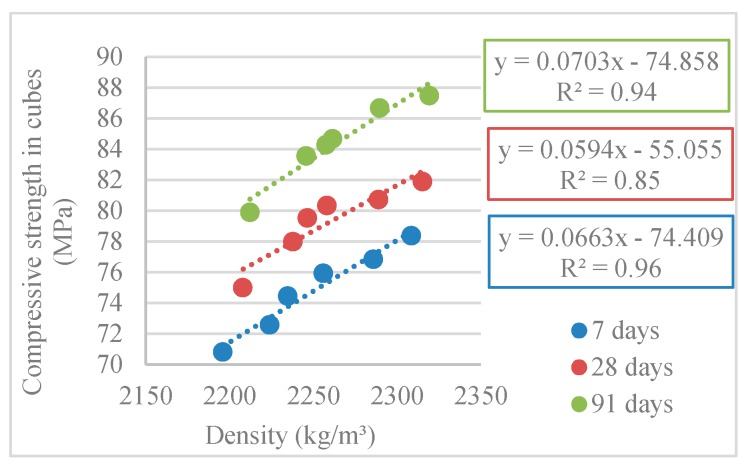
Correlation between compressive strength in cubes (15 cm × 15 cm × 15 cm) and density at 7, 28, and 91 days.

From Figure 16, a close correlation was found between the compressive strength and density (linear correlation with R^2^ = 0.96, R^2^ = 0.85 and R^2^ = 0.94).

#### 3.2.3. Compressive Strength (ϕ15 cm × 30-cm Cylinders)

**Figure 17:**
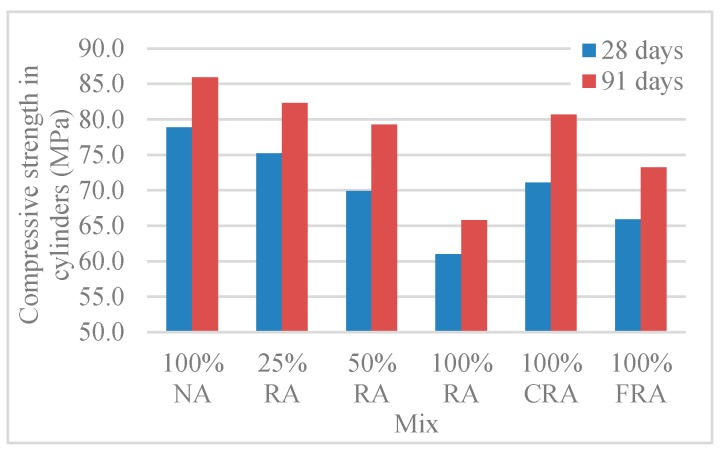
Compressive strength (ϕ15 cm × 30-cm cylinders) at 28 and 91 days.

Table 6 and Figure 17 show the results of compressive strength in ϕ15 cm × 30 cm cylinders (*f_cm,cyl_*).

The analysis of Table 6 and Figure 17 showed a reduction of compressive strength with the replacement of NA with RA (such as in cubes’ compressive strength, and for the same reason). The variations were between 4% and 23% and, as expected, the compressive strength increased with age.

**Figure 18:**
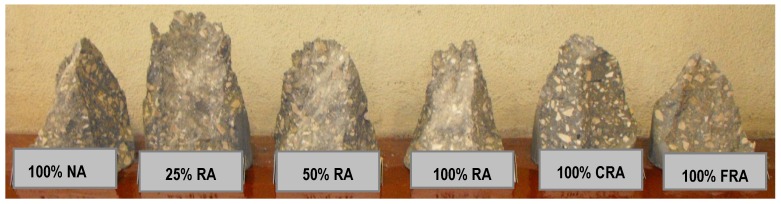
Failure surfaces presented by the different concrete mixes at 28 days.

Analysis of the failure surfaces obtained in cylindrical specimens revealed satisfactory failures (Figure 18) due to uniaxial compression, according to NP EN 12390-3 [46]. The type of failure obtained was explosive (as in cubic specimens). According to Parande [7], this type of failure is normal in HPC.

**Figure 19:**
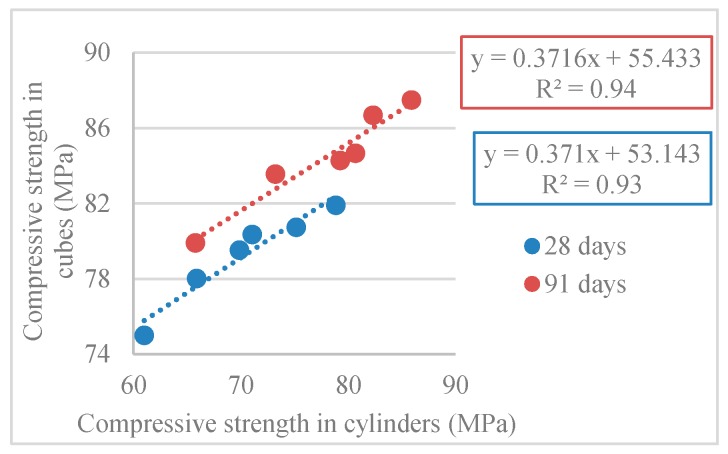
Compressive strength correlation among cubic/cylindrical specimens (28 and 91 days).

Table 7 and Figure 19 show the compressive strength overall correlation among cubic and cylindrical specimens.

At 28 and 91 days, the compressive strength in cylinders was, on average, 0.89 and 0.92 of that in cubes at 28 and 91 days, respectively (Table 7). In other words, the conversion factor of cubic specimens relative to cylindrical specimens was approximately 0.89 and 0.92 at 28 and 91 days. This factor is close to the values given in Table 7 of NP EN 206-1 [2], which range from 0.80 (class C8/10) to 0.87 (class C100/ 115) at 28 days.

Table 8 shows the characteristic strength values for cubes and cylinders specimens. The characteristic strength values (*f_ck_*) were obtained from Equation (1), present in Table 3.1 from Eurocode 2 [20]:

(1) $$f_{ck}=f_{cm}-8$$

Thus, a class was assigned according to the quality and safety control criteria specified in NP EN 206-1 [2]. As seen in Table 8, only the 100% RA mix belonged to strength class C50/60, while the rest of the mixes belonged to the class above, C55/67. According to NP EN 206-1 [2], all mixes were classified as HPC. The NP EN 206-1 standard considers that a mix classified as HPC must be of a strength class greater than C50/60.

#### 3.2.4. Splitting Tensile Strength

**Figure 20:**
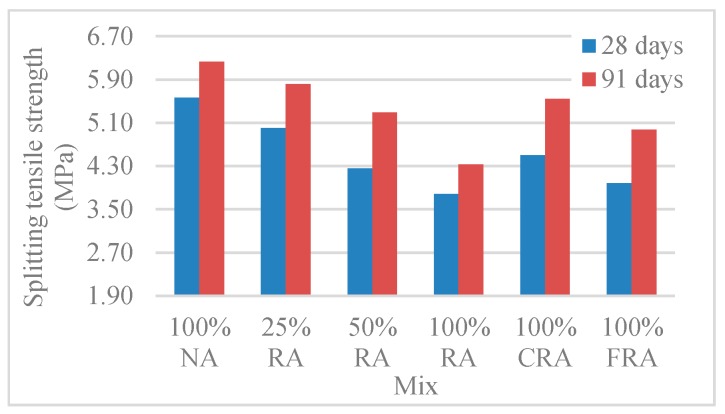
Splitting tensile strength (28 and 91 days).

Table 9 and Figure 20 show the results of the splitting tensile strength test on cylinders with ф15 cm × 30 cm (*f_ctm,sp_*). Figure 20 shows that the substitution of NA with RA was responsible for a decrease in splitting tensile strength. The lowest strength, 3.78MPa and 4.33MPa at 28 and 91 days, respectively, corresponded to the mix with 100% RA. The loss of strength in mixes with RA ranged from 7% to 32%. This is explained by the adhered mortar in RA [57]. Comparing SCC with 100% NA and SCC with 100% RA (at 28 days), Kou and Poon [59] and Modani and Mohitkar [58] achieved strength losses of 36% and 37%, respectively.

Figure 20 shows that the splitting tensile strength increased with age (as predicted). At 28 days, concrete achieved, on average, 78% of the strength at 91 days. Panda and Bal [57] obtained similar results, i.e., at 28 days, the splitting tensile strength reached 85% of the strength at 91 days.

**Figure 21:**
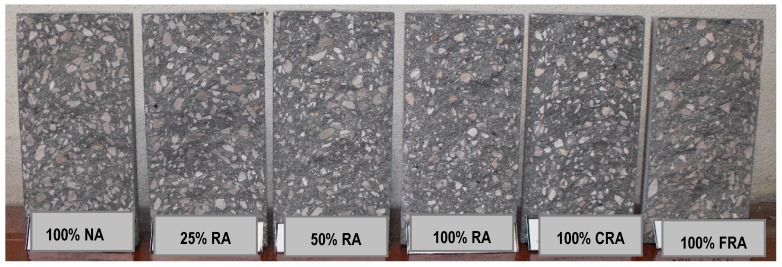
Failure surfaces for all mixes at 28 days.

**Figure 22:**
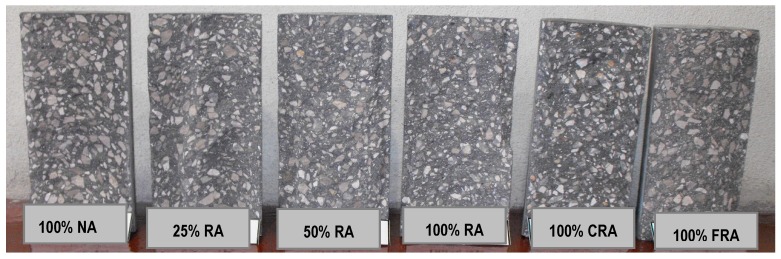
Failure surface for all mixes at 91 days.

This test made it possible to observe the failure surface of the test specimens. Thus, Figure 21 and Figure 22 show the typical failure surfaces (at 28 and 91 days). A uniform distribution of the coarse aggregate in the mixes was observed throughout the specimens, without particle agglomeration or any segregation or exudation. Along the failure surface, small air bubbles were observed, but they were not interconnected. These bubbles corresponded to the air trapped in HPSCC, as it did not undergo a vibration process.

Eurocode 2 [20] presents an equation that relates splitting tensile strength with compressive strength obtained in cylinders. Thus, for the calculation of splitting tensile strength ($f_{\mathrm{ctm},\mathrm{sp}}$) from the experimental values of cylinders compressive strength ($f_{\mathrm{cm},\mathrm{cyl}}$), Equation (2) from Table 3.1 of Eurocode 2 [20] was used:

(2) $$f_{\mathrm{ctm},\mathrm{sp}}=2.12\ln\left[1+\left(\frac{f_{\mathrm{cm},\mathrm{cyl}}}{10}\right)\right]/0.9\;(>C50/60)$$

In this equation, the axial tensile strength was considered to correspond to 90% of the splitting tensile strength, as suggested in Eurocode 2 [20].

Table 10 compares the tensile strength experimental results with the values obtained through the relationship defined in Eurocode 2 [20]. This shows a proximity between those pairs of values. Furthermore, part of the concrete mixes had tensile strength values slightly higher than those defined in Eurocode 2 [20].

**Figure 23:**
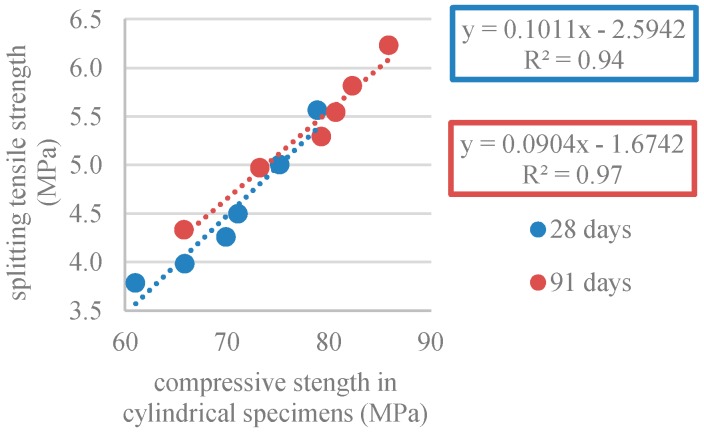
Splitting tensile strength and compressive strength correlation in cylindrical specimens (28 and 91 days).

Figure 23 shows the relationship between uniaxial compressive strength and splitting tensile strength. Considering the high coefficient of variation (R^2^ = 0.94 and R^2^ = 0.97), a strong correlation existed between these two properties.

#### 3.2.5. Secant Modulus of Elasticity

**Figure 24:**
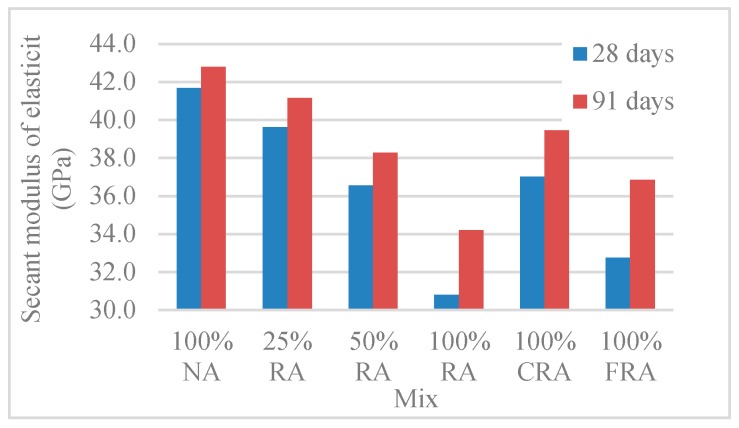
Secant modulus of elasticity results (28 and 91 days).

Table 11 and Figure 24 show the results obtained in the secant modulus of elasticity test (*E_cm_*).

There was a decrease of the modulus of elasticity as the incorporation of RA increased, with maximum variations of 26% and 20% at 28 and 91 days, respectively (for the 100% RA mix). It is noteworthy that Pereira-de-Oliveira et al. [54] achieved a 25% reduction to the 100% RA mix at 28 days.

The modulus of elasticity reduction is explained by the lower RA’s stiffness (compared to the NA’s), due to the presence of old mortar adhered to the aggregates and to the lower deformability of that old mortar [54]. Comparing the 100% NA mix with the 100% CRA mix, there was a reduction of 11% and 8% at 28 and 91 days, respectively. Under similar conditions, Uygunoğlu et al. [60] achieved a reduction of about 14% at 28 days. The authors justified this using the longer shape of CRA (compared to CNA) and the weaker bond between the cement matrix and CRA.

To calculate the modulus of elasticity ($E_{\mathrm{cm}}$), from the compressive strength experimental values in cylinders ($f_{\mathrm{cm},\mathrm{cyl}}$), Equation (3), proposed in Table 3.1 of Eurocode 2 [20], was used:

(3) $$E_{\mathrm{cm}}=22{\left[\left(f_{\mathrm{cm},\mathrm{cyl}}\right)/10\right]}^{0.3}$$

In Table 12, the experimental results of the modulus of elasticity are compared with those obtained through the relationship defined in Eurocode 2 [20].

Table 12 shows the proximity of the experimental modulus of elasticity with that obtained through the relationship defined in Eurocode 2 [20]. Most concretes mixes had modulus of elasticity values slightly lower than those defined in Eurocode 2 [20].

**Figure 25:**
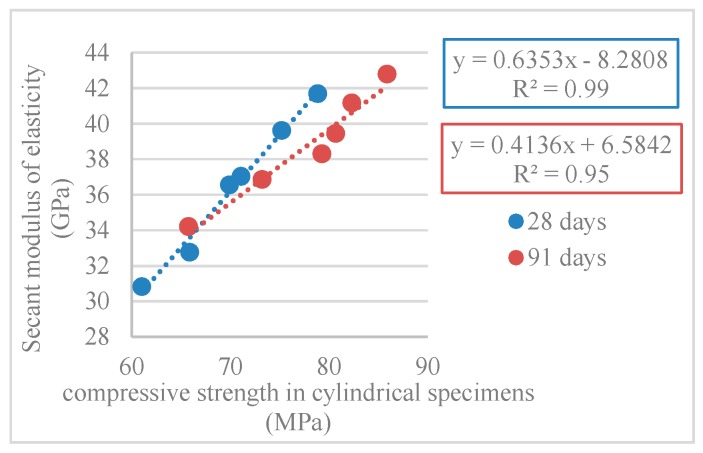
Secant modulus of elasticity and compressive strength correlation in cylindrical specimens (28 and 91 days).

**Figure 26:**
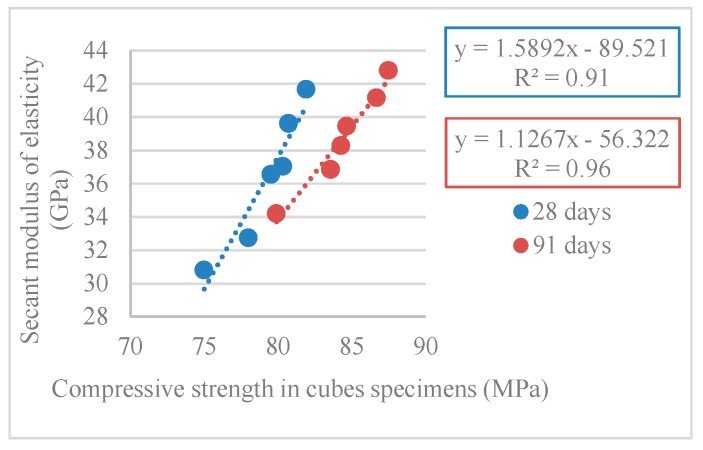
Secant modulus of elasticity and compressive strength correlation in cubes specimens (28 and 91 days).

Figure 25 and Figure 26 show the correlations obtained between the modulus of elasticity and compressive strength in cylindrical and cube specimens. R^2^ values of 0.99 and 0.95 (for compressive strength in cylinders) and 0.91 and 0.96 (for compressive strength in cubes) were obtained.

#### 3.2.6. Ultrasonic Pulse Velocity

**Figure 27:**
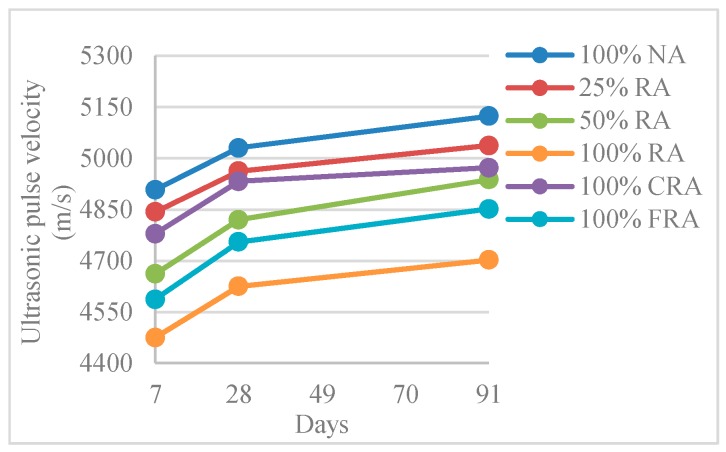
Ultrasonic pulse velocity (7, 28, and 91 days).

Table 13 and Figure 27 show the results obtained in the ultrasonic pulse velocity test (*V_usm,c_*).

The *V_usm,c_* decreased with the increased RA incorporation ratio. Thus, the reference mix (100% NA) had a higher *V_usm,c_*, reaching 5124 m/s at 91 days. On the other hand, compared with the 100% NA mix, the 100% RA mix had a lower *V_usm,c_* by 8%.

The reduction in *V_usm,c_* with the use of RA can be explained by the RA nature, i.e., higher porosity when compared to NA (due to the adhered old mortar).

Tuyan et al. [56] obtained results with the same trends, with 4% loss in pulse velocity for mixes with 60% replacement of NA with RA.

Malhotra [61] proposed a classification system for concrete quality as a *V_usm,c_* function where, with the values obtained in the present work (3660–4580 m/s range), we can classify all mixtures as “good”. With this classification, it was possible to estimate that none of the mixes had significant voids, volumes, or cracks that may have compromised its performance.

**Figure 28:**
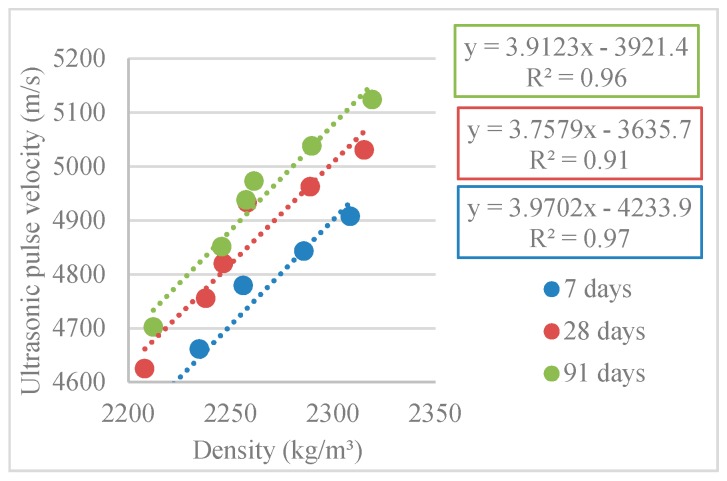
Density and ultrasonic pulse velocity correlation (7, 28, and 91 days).

Figure 28 shows the linear relationship between pulse velocity and density (R^2^ = 0.97; R^2^ = 0.91; R^2^ = 0.96). Thus, the denser the concrete, the faster the waves propagated (higher pulse velocity speed).

**Figure 29:**
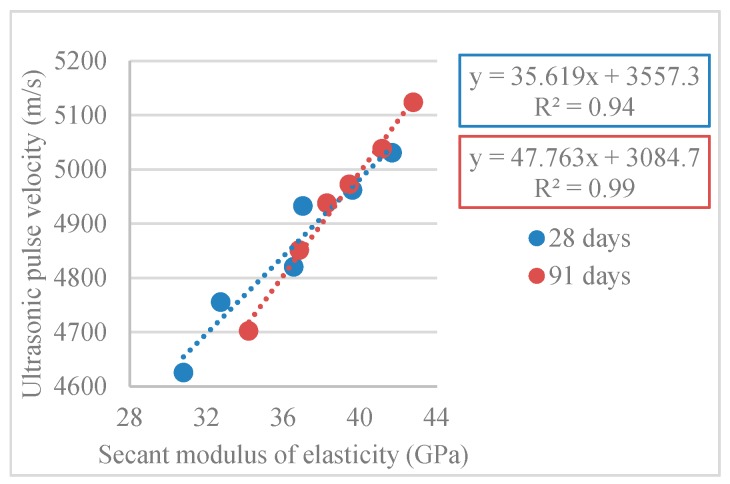
Secant modulus of elasticity and ultrasonic pulse velocity correlation (28 and 91 days).

Figure 29 shows the linear relationship between pulse velocity and modulus of elasticity (R^2^ = 0.94; R^2^ = 0.99).

These trends were confirmed by Bogas et al. [33], who stated that the main physical properties that influence ultrasonic pulse velocity are density and modulus of elasticity.

#### 3.2.7. Abrasion Resistance

**Figure 30:**
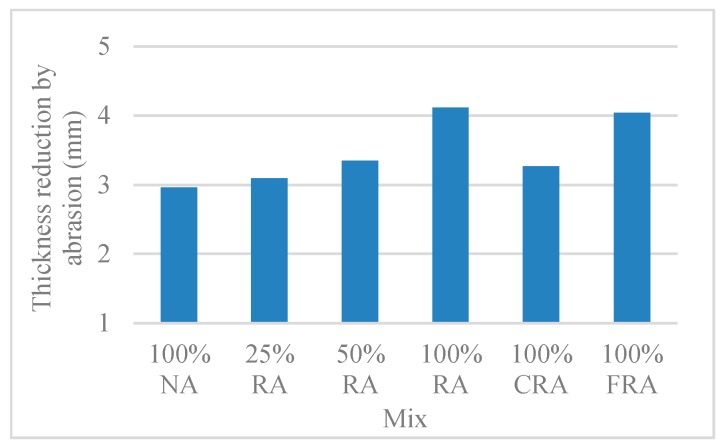
Abrasion resistance: Thickness reduction (91 days).

Table 14 and Figure 30 show the thickness reduction by abrasion results. Figure 30 shows a trend for a greater thickness reduction as the RA substitution ratio increases, which was consistent with the trends in other mechanical parameters studied (compressive strength and modulus of elasticity). The greatest thickness reduction (39%) occurred in the 100% RA mix. In the case of conventional concrete with 100% RA, Barbudo et al. [62] and Lotfi et al. [63] achieved thickness reductions of 17% and 40%, respectively. This is explained by the more porous nature of RA and the adhered old mortar.

Comparing the reference (100% NA) and 100% FRA mixes, a thickness reduction of 36% occurred. Pereira et al. [64] achieved thickness reductions of the same order of magnitude. For conventional concrete with RA, 21%, 37%, and 50% thickness reduction was achieved for concrete without S_p_, with conventional S_p_, and with high-range S_p_, respectively. This thickness reduction is explained by the increase in the effective w/c ratio observed in FRA concrete, as well as the higher porosity of RA.

#### 3.2.8. Shrinkage

**Figure 31:**
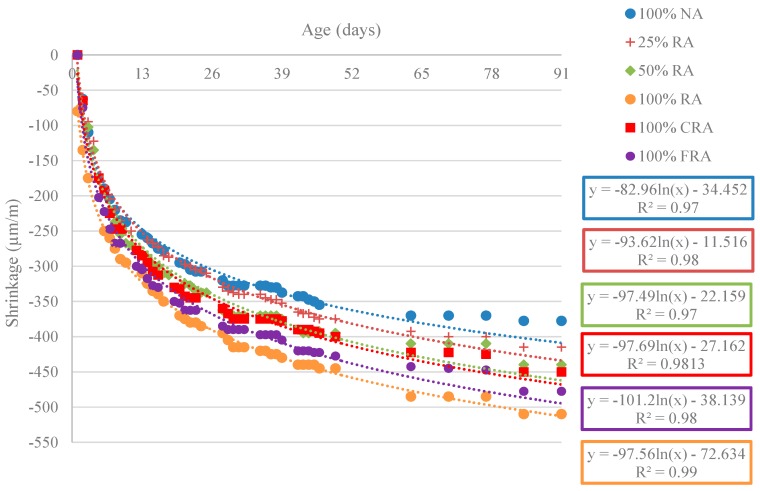
Evolution of shrinkage over 91 days.

Figure 31 shows the results obtained for the shrinkage evolution over 91 days ($\varepsilon_{cs}$).

For all mixes, Figure 31 shows that the shrinkage increased nonlinearly over time, as expected. Rapid growth was observed during the early days, which subsequently tended to stabilize. The use of logarithmic regressions led to very high correlation coefficients (R^2^) (between 0.97 and 0.99). Cartuxo et al. [65] found that the shrinkage increases analogously and obtained correlation coefficients greater than 0.95.

Replacing NA with RA led to a decrease in the performance of all mixes. Thus, the 100% RA mix showed the highest shrinkage strain value, with an increase over the reference SCC of 35% at 91 days. However, the values were well below those obtained by Kou and Poon [59], who recorded shrinkage increases of 166% (w/c = 0.53) and 103% (w/c = 0.44) for 100% RA replacement at 91 days. The authors justified these results with the RA higher porosity and consequent lower density that decreased their stiffness and ability to restrict strains.

Table 15 shows the shrinkage strains values at 7, 28, and 91 days. Thus, it is possible to analyze the behaviour of high-performance self-compacting concrete with recycled aggregates (HPSCCRA) in terms of shrinkage at young and older ages. The results show distinct behaviours over time.

Table 15 shows that shrinkage strain tended to increase with age (comparing RA mixes with the reference mix), e.g., 50% RA versus 100% NA showed increases in strain of 7%, 13%, and 15% at 7, 28, and 91 days, respectively. This phenomenon is explained by the internal curing carried out by RA, which allows the evaporation water to be compensated by existing water in RA. Thus, as long as water is available inside RA, the dimensional shrinkage variations are reduced [66].

**Figure 32:**
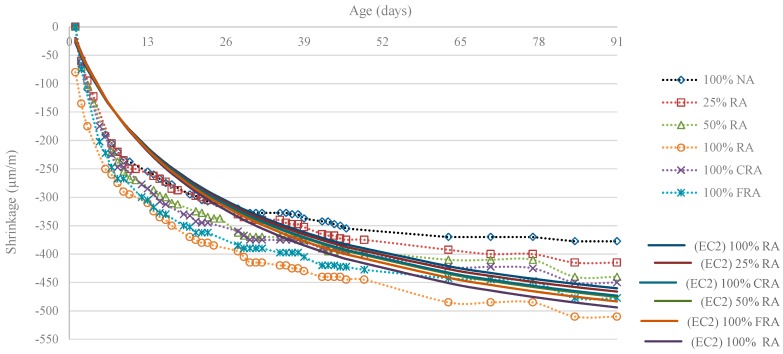
Evolution of shrinkage over 91 days: experimental values and based on EUROCODE 2 [20] values.

For the shrinkage strain calculation ($\varepsilon_{\mathrm{cs}}$) from the compressive strength experimental values in cylinders ($f_{\mathrm{cm}}$), the equation for conventional concrete’s shrinkage proposed in Eurocode 2 [20] was used. For the sake of readability, the total shrinkage strain values of the conventional mixes (estimated according to the equation proposed in part 1-1 (General rules and rules for buildings) of Eurocode 2 [20] and shown in Table 16 and Figure 32) only corresponded to the average stresses obtained by HPSCC produced in cylindrical specimens at 28 and 91 days. The seven-day values were calculated with a *f_cm,cyl_*/*f_cm,c_* ratio of approximately 0.82.

In Table 16 and Figure 32, the experimental results of shrinkage strain are compared with those obtained through the relationship defined in Eurocode 2 [20].

Figure 32 shows that the prediction model proposed by Eurocode 2 [20] presented shrinkage strains lower than the experimental strains (for example, at seven days, the difference between the experimental strain and the predicted strain through Eurocode 2 [20] was, on average, 85 µm/m). In contrast, at older ages (91 days), the Eurocode 2 prediction model [20] results generally tended to be close to the experimental results (for example, at 91 days, the difference between experimental strain and the predicted strain through Eurocode 2 [20] was, on average, 30 µm/m). For older ages, it was also found that the prediction model proposed by Eurocode 2 [20] tended to underestimate shrinkage strain (with the exception of the 100% RA mix).

Thus, the prediction model proposed by Eurocode 2 [20] cannot predict shrinkage strain at early ages, but tended to be similar in the long-term the strains. This was also found by Silva and de Brito [28].

The results of shrinkage strain agreed with those of the modulus of elasticity test. Mixes with RA had a lower modulus of elasticity than the reference mix (NA only). Compared to NA, the lower stiffness of RA decreased the overall stiffness of the mixes and thus increased the shrinkage strain.

**Figure 33:**
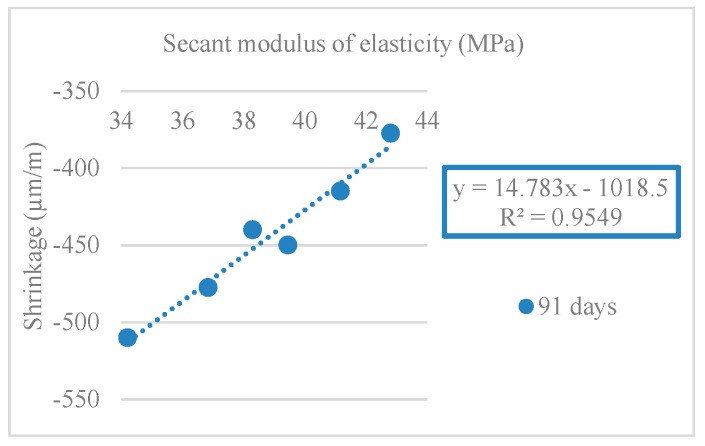
Shrinkage and secant modulus of elasticity correlation (91 days).

Figure 33 shows the relationship between the modulus of elasticity and shrinkage (R^2^ = 0.85).

**Figure 34:**
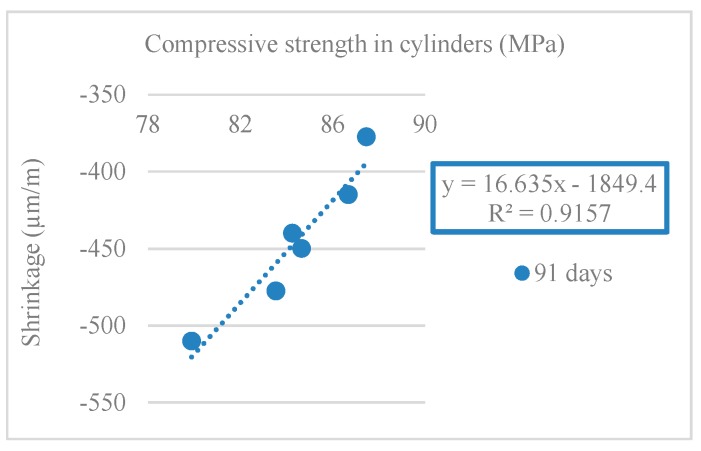
Shrinkage and compressive strength in cylinders correlation (91 days).

Figure 34 shows the correlation between shrinkage deformation and compressive strength at 91 days. The results led to the conclusion that there was a linear trend of shrinkage decrease with increasing compressive strength (R^2^ = 0.94).

#### 3.2.9. Creep

**Figure 35:**
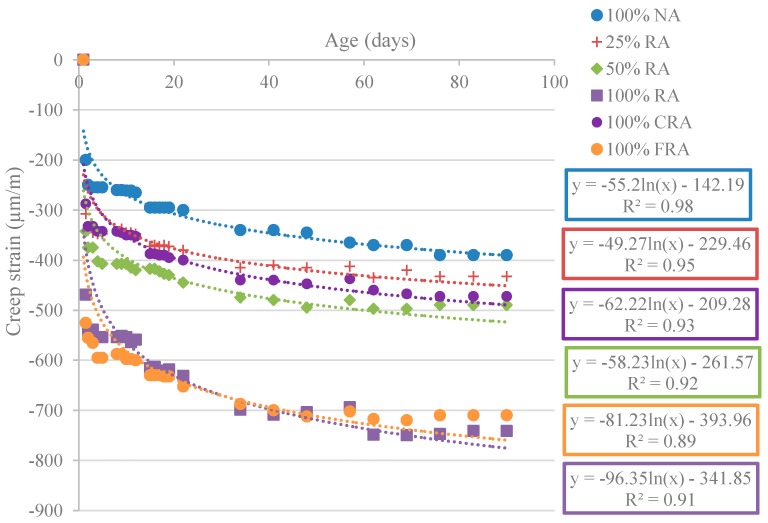
Creep strain over 91 days.

For the determination of creep strain, the concrete specimens were loaded at 28 days for 91 days. Figure 35 shows the results obtained for creep strain.

As with shrinkage, creep increased nonlinearly over time (Figure 35). The use of logarithmic regressions led to high correlation coefficients (R^2^) (between 0.85 and 0.95). The creep phenomenon took place mainly at early ages, translating into a rapid growth of the strain in the early days which tended to stabilize afterward.

The obtained results showed that the creep strain increased with the substitution ratio of NA with RA, leading to a reduction in concrete performance. The increase in creep with increasing RA replacement was due to the reduction of the elastic modulus of RA relative to NA. RA have a lower stiffness and therefore a lower ability to resist the creep phenomenon.

Mixes with 50% RA and 100% CRA showed an increase in creep strain of 34% and 30%, respectively (compared to 100% NA). Manzi et al. [67] achieved a creep strain increase of 15% for concretes with 50% RA incorporation. Domingo-Cabo et al. [68] observed a 51% increase in creep strain for 100% CRA mix. The authors justified this behaviur with the smaller modulus of elasticity of RA compared to NA’s.

Table 17 shows the creep strain coefficients for all mixes. All mixes had very close creep strain coefficients.

**Figure 36:**
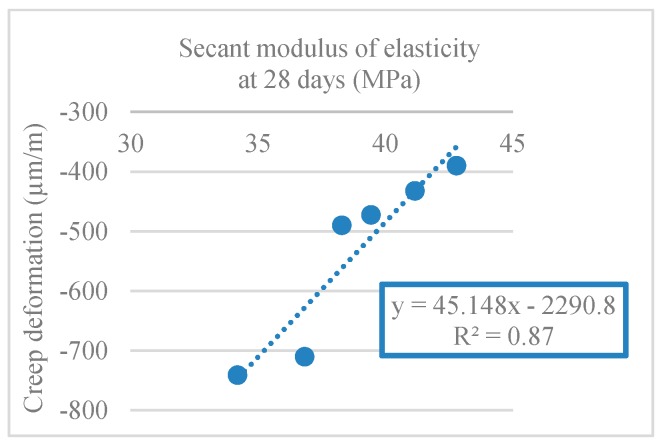
Creep strain and secant modulus of elasticity correlation (28 days).

**Figure 37:**
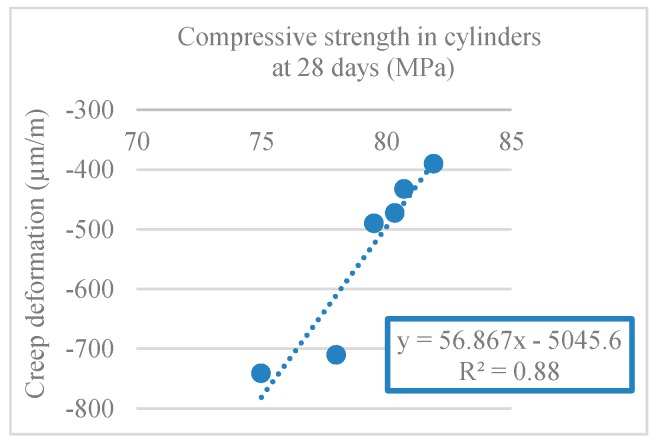
Creep strain and compressive strength in cylinders correlation (28 days).

Figure 36 shows a linear relationship between creep strain and modulus of elastic (R^2^ = 0.87). In Figure 37, there is a linear relationship between creep and compressive strength (R^2^ = 0.88).

### 3.3. Global Analysis of Results

Replacing NA with AR caused decreased compressive strength and splitting tensile strength. This is explained by the poorer quality of RA due to their adhered mortar, which is responsible for increasing the porosity and cracking of RA, thus weakening the transition zone connections between RA and the new cement binder. At 28 days, there was a decrease of 1–8% in cubic compressive strength, 5–23% in cylindrical compressive strength, and 10–32% on splitting tensile strength.

According to the classification of NP EN 206-1 [2], the 100% RA mix belonged to class strength C50/60 and the remaining mixes belonged to a higher class, C65/67. Thus, all mixes can be classified as high performance (HPSCC).

There was a decrease in the modulus of elasticity with the increase of RA incorporation, which is explained by the lower RA’s stiffness (compared to NA) due to the presence of adhered old mortar in RA and to the lower deformability of the mortar.

The lower RA stiffness decreased the overall concrete stiffness and thus increased the shrinkage and creep strain. The shrinkage and creep increases at 91 days were higher than those at 7 days due to the internal healing phenomenon triggered by RA.

Ultrasonic pulse velocity decreased with increasing RA replacement ratio. This decrease was small (1% to 8%, at 28 days) and is explained by RA’s quality, i.e., higher porosity than NA.

There was an increase of the thickness reduction by abrasion as the RA replacement ratio increased (at 28 days a thickness reduction up to 39% occurred).

Eurocode 2 [20] provides equations to estimate the properties of conventional concrete based on experimental results of compressive strength in cylinders obtained at 28 days. Thus, these equations were used to estimate other properties (splitting tensile strength, secant modulus of elasticity, and shrinkage strain) and were compared with the experimental results.

Comparing the experimental results with those obtained through the Eurocode 2 [20] equations, the prediction models tended to provide values reasonably close to those obtained experimentally.

## 4. Concluding Remarks

This work intended to analyze the experimental results obtained in the production of HPSCC with recycled aggregates and to validate its viability regarding mechanical properties. Six types of HPSCC were produced incorporating different amounts of RA. Five replacement ratios of FRA and CRA (FRA/CRA) were considered (25/25%; 50/50%; 100/100%; 0/100%, and 100/0%) in addition to a reference mix (FRA/CRA) of 0/0%.

The results of the fresh-state properties showed that all mixes fulfilled the workability parameters required by NP EN 206-9 [39]. Thus, all the produced mixes had the required characteristics for SCC.

The results showed that the different mixes incorporating RA presented coherent performance variations between themselves and when compared with results of other authors.

For the maximum replacement ratio (100% RA), the compressive strength and modulus of elasticity showed performance losses of no more than 26%.

The 25% RA mix had the best performance, with mechanical properties losses of less than 10%.

For all mechanical properties under analysis, the 50% RA and 100% CRA mixes had similar performances. Additionally, the first tended to have worse results than the second.

The 100% FRA mix presented a less favorable performance than the 100% RA mix. Comparing these two mixes, both had 100% FRA instead of FNA, differing only in the nature of coarse aggregates (with 100% CNA and 100% CRA respectively). Therefore, the differences may be justified by the nature of CA and the water they absorbed.

There is a need to correct the amounts of mixing water due to the high-water absorption of RA. This means that, particularly for CRA, the mixes can retain considerable amounts of water during the initial mixing phase. This water, which is not used for workability or any hydration process at an early age, is available for later release to contribute to the later reaction of FA with calcium oxide or calcium hydroxide (products resulting from cement hydration).

With these results, it is possible to confirm the viability of producing HPSCC with recycled aggregates from the precast industry. The mixes with incorporation ratios of 25% RA, 50% RA, and 100% CRA are viable and have very interesting performance. The other mixes also perform well, with a performance reduction of less than 25%.

## Figures and Tables

**Table 1:** Chemical composition of the raw materials (%).

Chemical Composition *	CEM I	FA	LF	SF
Al_2_O_3_	5.24	24.7	0.13	0.54
CaCO_3_	-	-	98.35	-
CaO	62.71	2.63	-	0.43
Cl^−^	0.01	<0.01	-	-
Fe_2_O_3_	3.17	5.40	0.03	1.15
K_2_O	-	1.11	0.02	0.86
MgO	2.23	1.01	0.40	0.43
Na_2_O	-	0.89	-	0.29
SiO_2_	19.59	54.70	0.30	93.67
SO_3_	3.13	1.38	-	0.25
TiO_2_	-	-	0.01	-
Insoluble residue	1.37	-	-	-
Loss of ignition	2.94	5.10	43.80	2.54
Surface area (BET) (m^2^/kg) (NP EN 196-6 [30])	325	430	456	15000

* The chemical composition of the raw materials was provided by the respective producers. CEM I: Cement type I 52.5 R; FA: Fly ash; LF: Limestone filer; SF: Silica fume.

**Table 2:** Grading of the raw materials.

Particle Size, in Microns *	Passing (%)			
	CEM I	FA	LF	SF
1000	100	100	100	100
100	98	96	60	100
10	38	45	20	80
1	5	2	0	10
0.1	0	0	0	2

* The grading of the raw materials was provided by the respective producers.

**Table 3:** Mix proportions of the high-performance self-compacting concrete with recycled aggregates (HPSCCRA).

Mix Proportions (kg/m^3^)	100% NA	25% RA	50% RA	100% RA	100% CRA	100% FRA
CEM I 52.5 R (C)	437					
Fly ash (FA)	145					
Limestone filler (LF)	29					
Silica fume (SF)	27					
Superplasticizer (S_p_)	8					
Water (W)	193					
Sand_0/2_ (FNA_0/2_)	162	122	81	-	162	-
Sand_0/4_ (FNA_0/4_)	484	363	242	-	484	-
FRA	-	145	290	581	-	581
Gravel_1_ (CNA_1_)	389	292	195	-	-	389
Gravel_2_ (CNA_2_)	398	299	199	-	-	398
CRA	-	184	369	737	737	-
W/C ratio	0.442					
W/CM ratio	0.317					
W/FM ratio	0.303					

W/C, water/cement; W/CM, water/cementitious materials; W/FM, water/fine materials.

**Table 4:** Density at 7, 28, and 91 days.

Mix	7 days			28 days			91 days		
	Density	S.D.	∆_100%_ NA	Density	S.D.	∆_100%_ NA	Density	S.D.	∆_100%_ NA
	(kg/m^3^)		(%)	(kg/m^3^)		(%)	(kg/m^3^)		(%)
100% NA	2308.5	4.9	0.0	2315.3	12.9	0.0	2319.4	14.1	0.0
25% RA	2285.8	9.1	−1.0	2289.0	14.7	−1.1	2289.7	11.4	−1.3
50% RA	2234.8	6.3	−3.2	2246.5	9.9	−3.0	2257.7	2.5	−2.7
100% RA	2196.0	2.5	−4.9	2208.0	8.8	−4.6	2212.3	11.3	−4.6
100% CRA	2256.1	3.6	−2.3	2258.3	7.5	−2.5	2261.5	5.6	−2.5
100% FRA	2223.9	14.5	−3.7	2238.0	7.7	−3.3	2245.7	6.4	−3.2

**Table 5:** Compressive strength (15 cm × 15 cm × 15 cm cubes) at 7, 28, and 91 days.

Mix	7 days				28 days				91 days			
	*f_cm,c,7d_*	S.D.	C.V.	∆_100%_ NA	*f_cm,c,28d_*	S.D.	C.V.	∆_100%_ NA	*f_cm,c,91d_*	S.D.	C.V.	∆_100%_ NA
	(MPa)		(%)	(%)	(MPa)		(%)	(%)	(MPa)		(%)	(%)
100% NA	78.4	0.3	0.4	0.0	81.9	2.5	3.1	0.0	87.5	0.5	0.6	0.0
25% RA	76.8	2.8	3.6	−2.0	80.7	0.4	0.5	−1.4	86.7	1.3	1.5	−0.9
50% RA	74.5	0.6	0.8	−5.0	79.5	2.5	3.1	−2.9	84.3	2.1	2.5	−3.7
100% RA	70.8	1.4	2.0	−9.7	75.0	0.5	0.7	−8.4	79.9	1.9	2.4	−8.7
100% CRA	75.9	1.2	1.6	−3.1	80.3	5.9	7.3	−1.9	84.7	1.0	1.2	−3.2
100% FRA	72.6	0.2	0.3	−7.4	78.0	2.2	2.8	−4.8	83.6	3.1	3.7	−4.5

**Table 6:** Compressive strength (ϕ15 cm × 30-cm cylinders) at 28 and 91 days.

Mix.	28 days			91 days		
	*f_cm,cyl,28d_*	S.D.	∆_100%_ NA	*f_cm,cyl,91d_*	S.D.	∆_100%_ NA
	(MPa)		(%)	(MPa)		(%)
100% NA	78.9	1.1	0.0	85.9	1.4	0.0
25% RA	75.2	1.1	−4.7	82.3	2.8	−4.2
50% RA	69.9	2.0	−11.4	79.3	3.0	−7.7
100% RA	61.0	1.8	−22.6	65.8	1.0	−23.4
100% CRA	71.1	2.1	−9.9	80.7	1.1	−6.1
100% FRA	65.9	1.2	−16.5	73.3	1.3	−14.7

**Table 7:** Compressive strength correlation among cubic/cylindrical specimens (28 and 91 days).

Mix.	28 days			91 days		
	*f_cm,c,28d_*	*f_cm,cyl,28d_*	*f_cm,cyl,28d_/f_cm,c,28d_*	*f_cm,c,91d_*	*f_cm,cyl,91d_*	*f_cm,cyl,91d_/f_cm,c,91d_*
	(MPa)			(MPa)		
100% NA	81.9	78.9	0.96	87.5	85.9	0.98
25% RA	80.7	75.2	0.93	86.7	82.3	0.95
50% RA	79.5	69.9	0.88	84.3	79.3	0.94
100% RA	75.0	61.0	0.81	79.9	65.8	0.82
100% CRA	80.3	71.1	0.89	84.7	80.7	0.95
100% FRA	78.0	65.9	0.84	83.6	73.3	0.88

**Table 8:** NP EN 206-1 [2] compressive strength classes.

Mix.	Experimental Values				NP EN 206-1 Values		
	*f_cm,cyl,28d_*	*f_cm,c,28d_*	*f_ck,cyl_*	*f_ck,c_*	Class	*f_ck,cyl_*	*f_ck,c_*
	(MPa)					(MPa)	
100% NA	78.9	81.9	70.9	73.9	C55/67	55	67
25% RA	75.2	80.7	67.2	72.7	C55/67	55	67
50% RA	69.9	79.5	61.9	71.5	C55/67	55	67
100% RA	61.0	75.0	53.0	67.0	C50/60	50	60
100% CRA	71.1	80.3	63.1	72.3	C55/67	55	67
100% FRA	65.9	78.0	57.9	70.0	C55/67	55	67

**Table 9:** Splitting tensile strength (28 and 91 days).

Mix.	28 days				91 days			
	*f_ctm,sp,28d_*	S.D.	C.V.	∆_100%_ NA	*f_ctm,sp,91d_*	S.D.	C.V.	∆_100%_ NA
	(MPa)		(%)	(%)	(MPa)		(%)	(%)
100% NA	5.6	0.5	8.9	0.0	6.2	0.1	1.6	0.0
25% RA	5.0	0.4	8.0	−10.1	5.8	0.3	5.2	−6.7
50% RA	4.3	0.5	11.6	−23.4	5.3	0.4	7.5	−15.1
100% RA	3.8	0.7	18.4	−32.0	4.3	0.1	2.3	−30.5
100% CRA	4.5	0.1	2.2	−19.2	5.5	0.1	1.8	−11.0
100% FRA	4.0	0.4	10.0	−28.4	5.0	0.4	8.0	−20.2

**Table 10:** Splitting tensile strength and compressive strength correlation according to Eurocode 2 [20] (28 and 91 days).

Mix.	28 days			91 days		
	Average Compressive Strength	Average Splitting Tensile Strength		Average Compressive Strength	Average Splitting Tensile Strength	
	*f_cm,cyl,28d_*	EC2 Value	Experimental Values	*f_cm,cyl,91d_*	EC2 Value	Experimental Values
	(MPa)			(MPa)		
100% NA	78.9	4.6	5.6	85.9	4.8	6.2
25% RA	75.2	4.5	5.0	82.3	4.7	5.8
50% RA	69.9	4.4	4.3	79.3	4.6	5.3
100% RA	61.0	4.2	3.8	65.8	4.3	4.3
100% CRA	71.1	4.4	4.5	80.7	4.7	5.5
100% FRA	65.9	4.3	4.0	73.3	4.5	5.0

**Table 11:** Secant modulus of elasticity results (28 and 91 days).

Mix.	28 days				91 days			
	*E_cm,28d_*	S.D.	C.V.	∆_100%_ NA	*E_cm,91d_*	S.D.	C.V.	∆_100%_ NA
	(MPa)		(%)	(%)	(MPa)		(%)	(%)
100% NA	41.7	2.2	5.3	0.0	42.8	1.4	3.3	0.0
25% RA	39.6	1.5	3.8	−4.9	41.2	1.9	4.6	−3.8
50% RA	36.6	0.7	1.9	−12.3	38.3	0.2	0.5	−10.5
100% RA	30.8	6.3	20.5	−26.1	34.2	0.2	0.6	−20.1
100% CRA	37.0	0.6	1.6	−11.2	39.5	0.8	2.0	−7.8
100% FRA	32.8	0.1	0.3	−21.4	36.8	1.0	2.7	−13.9

**Table 12:** Secant modulus of elasticity and compressive strength correlation according to Eurocode 2 [20] in cylindrical specimens (28 and 91 days).

Mix.	28 days			91 days		
	Average Compressive Strength	Secant Modulus of Elasticity		Average Compressive Strength	Secant Modulus of Elasticity	
	*f_cm,cyl,28d_*	EC2 Value	Experimental Values	*f_cm,cyl,91d_*	EC2 Value	Experimental Values
	(MPa)	(GPa)		(MPa)	(GPa)	
100% NA	78.9	40.9	41.7	85.9	41.9	42.8
25% RA	75.2	40.3	39.6	82.3	41.4	41.2
50% RA	69.9	39.4	36.6	79.3	40.9	38.3
100% RA	61.0	37.9	30.8	65.8	38.7	34.2
100% CRA	71.1	39.6	37.0	80.7	41.2	39.5
100% FRA	65.9	38.7	32.8	73.3	40.0	36.8

**Table 13:** Ultrasonic pulse velocity (7, 28, and 91 days).

Mix.	7 days			28 days			91 days		
	*V_usm,c,7d_*	S.D.	∆_100%_ NA	*V_usm,c,28d_*	S.D.	∆_100%_ NA	*V_usm,c,91d_*	S.D.	∆_100%_ NA
	(m/s)		(%)	(m/s)		(%)	(m/s)		(%)
100% NA	4907.6	53.0	0.0	5030.7	4.0	0.0	5123.8	58.6	0.0
25% RA	4843.0	74.7	−1.3	4961.9	69.6	−1.4	5037.6	46.2	−1.7
50% RA	4623.3	19.7	−5.8	4819.8	29.9	−4.2	4937.8	12.5	−3.6
100% RA	4474.7	18.0	−8.4	4624.9	17.2	−8.1	4702.3	30.6	−8.2
100% CRA	4779.3	59.5	−2.6	4932.4	11.3	−2.0	4972.4	26.2	−3.0
100% FRA	4587.2	16.2	−6.5	4755.2	5.8	−5.5	4851.5	64.9	−5.3

**Table 14:** Abrasion resistance: Wear depth (91 days).

Mix.	Wear Depth	S.D.	∆_100%_ NA
	(mm)		(%)
100% NA	3.0	0.1	0.0
25% RA	3.1	0.2	4.5
50% RA	3.3	0.1	12.8
100% RA	4.1	0.1	38.6
100% CRA	3.3	0.1	10.2
100% FRA	4.0	0.3	36.2

**Table 15:** Shrinkage (7, 28, and 91 days).

Mix.	7 Days		28 Days		91 Days	
	$\varepsilon_{cs,7d}$	∆_100%_ NA	$\varepsilon_{cs,28d}$	∆_100%_ NA	$\varepsilon_{cs,91d}$	∆_100%_ NA
	(µm/m)	(%)	(µm/m)	(%)	(µm/m)	(%)
100% NA	−205.0	0.0	−320.0	0.0	−377.5	0.0
25% RA	−205.0	0.0	−330.0	3.1	−415.0	9.9
50% RA	−220.0	7.3	−362.5	13.3	−440.0	16.6
100% RA	−260.0	26.8	−395.0	23.4	−510.0	35.1
100% CRA	−225.0	9.8	−360.0	12.5	−450.0	19.2
100% FRA	−247.5	20.7	−385.0	20.3	−477.5	26.5

**Table 16:** Evolution of shrinkage: Experimental values and based on Eurocode 2 [20] as a function of compressive strength at 7, 28, and 91 days.

Mix.	7 Days		28 Days		91 Days	
	EC2 Value	$\varepsilon_{cs,7d}$	EC2 Value	$\varepsilon_{cs,28d}$	EC2 Value	$\varepsilon_{cs,91d}$
	(µm/m)		(µm/m)		(µm/m)	
100% NA	−142.5	−205.0	−316.5	−320.0	−460.2	−377.5
25% RA	−142.0	−205.0	−319.4	−330.0	−466.0	−415.0
50% RA	−141.5	−220.0	−324.3	−362.5	−475.4	−440.0
100% RA	−141.5	−260.0	−334.4	−395.0	−493.9	−510.0
100% CRA	−141.6	−225.0	−323.2	−360.0	−473.2	−450.0
100% FRA	−141.4	−247.5	−328.6	−385.0	−483.3	−477.5

**Table 17:** Creep strain coefficient (91 days).

Mix.	Creep at 91 days (Without Shrinkage)	*E_cm,91d_*	Instantaneous Creep	Final Creep	Creep Coefficient
	(µm/m)	(MPa)	(µm/m)	(µm/m)	
100% NA	−3.90 × 10^−4^	41682	2.40 × 10^−4^	1.50 × 10^−4^	0.63
25% RA	−4.33 × 10^−4^	39622	2.52 × 10^−4^	1.80 × 10^−4^	0.71
50% RA	−4.90 × 10^−4^	36556	2.74 × 10^−4^	2.16 × 10^−4^	0.79
100% RA	−7.41 × 10^−4^	30806	3.25 × 10^−4^	4.17 × 10^−4^	1.28
100% CRA	−4.73 × 10^−4^	37032	2.70 × 10^−4^	2.02 × 10^−4^	0.75
100% FRA	−7.10 × 10^−4^	32751	3.05 × 10^−4^	4.05 × 10^−4^	1.33
